# Utilizing multiplex fluor LAMPs to illuminate multiple gene expressions *in situ*

**DOI:** 10.1371/journal.pone.0223333

**Published:** 2019-10-04

**Authors:** Diona Podushkina, Nick W. West, Edward M. Golenberg

**Affiliations:** Department of Biological Sciences, Wayne State University, Detroit, Michigan, United States of America; Clemson University, UNITED STATES

## Abstract

*In situ* gene expression detection is the best way to determine temporal and spatial differences in gene expression. However, *in situ* hybridization procedures are inherently difficult to execute and typically suffer from degradation of sample tissues, limited sensitivity to genes with low expression, high background, and limitation to single gene detections. We propose to utilize an isothermal gene amplification technique, LAMP (Loop-Mediated Isothermal Amplification), to solve these problems in a novel way. LAMP greatly amplifies the signal of expressed genes and can use multiple sets of primers and different fluorescent-labeled probes to produce multiplex gene detection. LAMP is a rapid, isothermal reaction that reduces the handling and degradation of tissue by cutting down on the washing steps required by other methods. Using this technique, we have successfully amplified 3 target genes, have produced positive fluorescent *in situ* results simultaneously for two genes. We have also demonstrated that LAMP can be used to exploit standard NBT/BCIP (nitro-blue tetrazolium chloride/5-bromo-4-chloro-3'-indolyphosphate p-toluidine salt) detection of single expression. *In situ* LAMP is a robust and applicable method that can be exploited for detection of gene expression in plant species, as well as in animals and bacteria.

## Introduction

*In situ* hybridization is the best way to detect temporal and spatial differences in gene expression in complex tissues and organs and is widely used across a variety of fields within biology. In cellular and developmental biology, it is used for gene mapping, gene expression, cytogenetics, and developmental studies [1–6]. In public health and medical fields, it is used to detect both viral and bacterial pathogens, to monitor novel or abnormal gene expression in tumor tissues, and to diagnose genetic or developmental abnormalities prenatally [5, 7, 8]. *In situ* hybridization uses labeled oligo-nucleotides to bind or hybridize to complementary target RNA or DNA sequences [2, 7, 8]. The bound, labeled oligo-nucleotides, or probes, are then detected by a variety of methods. Typically, tissue sections or whole organisms are challenged with labeled antisense RNA sequence probes that are designed to bind to specific genes or gene transcripts. Under appropriate conditions, the antisense RNA sequences hybridize uniquely to their complementary sense mRNA transcripts where they are produced in the tissues. These labeled nucleic acid probes can be detected by radioactive exposure when radiolabeled, by secondary deposition of a colored substance when incorporated with biotin or digoxygenin, or by fluorescence when attached to fluorophores. When the labeled probes are detected, a fine scale understanding of where and at what stage specific genes are expressed can be determined.

Although *in situ* hybridization is a commonly used technique, it has several limitations. The first of these limitations is the difficulty to detect low or limited signals of expression within the tissue [2]. Some methods have been proposed to tackle this problem. These include both pre- and post-hybridization amplification steps. Three common methods of pre-hybridization amplification are *in situ* PCR, primed *in situ* labeling (PRINS), and *in situ* transcription [2]. *In situ* PCR utilizes a polymerase chain reaction through the addition of reverse transcriptase and DNase to the standard *in situ* hybridization reaction [4]. Although *in situ* PCR can be used to amplify genes with low expression, it has very low efficiency and the results are hard to reproduce [2, 4]. Additionally, it requires specially designed equipment and the repeated exposure to high temperatures contributes to sample damage, often leading to low morphological integrity. PRINS is another single-step amplification method which uses Taq DNA Polymerase to incorporate labeled nucleotides into an elongating DNA strand [2, 3]. PRINS has a fast reaction time and improves *in situ* sensitivity but it requires more advanced incubation equipment and better quality samples. It is also not able to detect multiple genes at once [2, 3]. *In situ* transcription is a similar method to PRINS with similar limitations [2, 5]. It is performed through the hybridization of a target specific complimentary oligonucleotide which acts as an initiator for reverse transcription [2, 5]. Detection of the produced cDNA activity is obtained through the incorporation of radiolabeled deoxynucleotides during the transcription process [5]. The use of radiolabeling creates the limitations of long exposure times and low resolution in final products [5].

Amplification after hybridization is another way to increase signal. Two such methods are catalyzed reporter deposition (CARD) and branched DNA technology. CARD created signal amplification through the deposition of an activated biotinylated tyramine by a catalyzing reporter enzyme [2]. CARD produces robust amplification of low signals; however, it can also produce amplified background signal and is mainly optimized for protein immunoassays rather than amplification of detected RNA transcripts within tissue samples [2]. Branched DNA technology is another method of post-hybridization amplification. It uses sequential hybridization of oligonucleotide probes to amplify the signal of the target rather than the target itself. The sequential washes have the disadvantage of degrading the tissue and this method is again optimized for immunoassays. These limitations make it unusable for spatial and temporal detection of gene expression in fragile tissue samples.

Colorimetric detection of labeled hybridized probes (CISH) is the most common *in situ* hybridization technique used. In this method biotin or digoxygenin labeled probes are used to detect target DNA regions [7, 8]. Biotin labeled probe detection is done through the use of streptavidin conjugated with horseradish peroxidase (HRP) or an anti-biotin labeled alkaline phosphatase (AP) enzyme that hydrolyzes BCIP and in turn is oxidized by NBT to produce an insoluble brown substrate [7]. Digoxygenin labeled probe detection works in a similar way but instead uses an anti-digoxygenin coupled AP enzyme. The major limitations of this colorimetric approach are that common colorimetric protocols generally cannot be used to detect more than a single gene expression pattern at a time except when done sequentially, and its sensitivity to detect low level of gene expression is limited.

Simultaneous, multiple gene detection is desirable for quickly determining the differential expression of selected genes in a single tissue sample. A modification of the standard colorimetric *in situ* hybridization technique that permits simultaneous multiple gene detection is Fluorescence *In situ* Hybridization (FISH). Individual gene probes are labeled with specific fluorophores instead of biotin or digoxygenin^7^. After hybridization and washing, hybridized probes can be directly visualized with an epifluorescent or confocal microscope [1]. However, FISH is not very time effective due to the extra hybridization and counter-staining steps and produces a limited hard-to-detect signal for genes with low expression [1]. The protocol steps must be optimized for each new probe further increasing the difficulty level of the procedure [1].

As mentioned previously, *in situ* hybridization can often produce large amounts of background, making it hard to differentiate between the real expression of the genes and false-positive background signal, especially for genes with low expression patterns. Troubleshooting steps such as increasing the hybridization time, increasing the number of hybridizations, or increasing the probe detection may increase the intended signal but will also often lead to increased background without increasing the signal.

**Fig 1:**
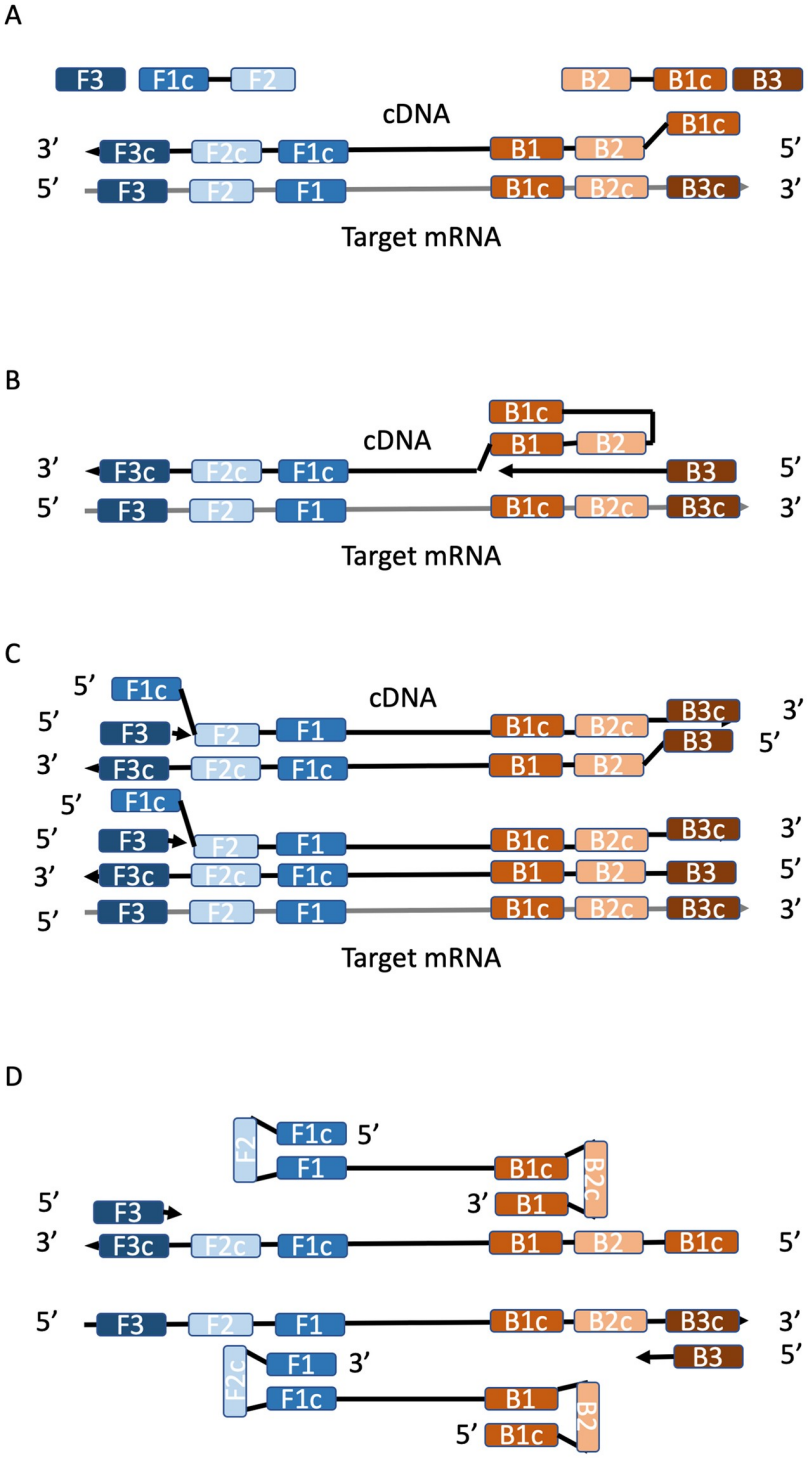
LAMP process with RNA template. A. Six target sequences (F1, F2, F3, B1, B2, and B3) are selected to amplify specific mRNA. Primers are designed such that outer F3 and B3 primers bind to the complementary sequences in the template RNA/DNA. Forward and Back Internal Primers, FIP and BIP, respectively, are designed such that the 3’ ends of the primers bind to the F2 and B2 complementary template sites, and the 5’ ends of the primers reverse complement the F1 and B1 sequences, F1c and B1c, respectively. B. The mRNA is reverse transcribed via a RT DNA Polymerase producing an RNA-DNA hybrid utilizing the BIP primer. C. The outer primer (B3) binds to the RNA template and initiates polymerization via strand displacing DNA Polymerase until the first cDNA strand is completely displaced. The 5’ end of the displaced strand forms a loop by complementary binding of the B1c (on the primer) and B1 sequence. C. The FIP primer binds to the new cDNA strand at the F2c site, initiating polymerization of a forward strand. It in tern is displaced by synthesis initiated by an outer primer (F3), allowing the F1c region to bind to the F1 sequence on the same strand, D. creating a self-hybridizing loop structure. The annealing and displacement process are then repeated forming concatemers of dumbbell shaped products [9–11].

To overcome these limitations, we have developed a novel technique for *in situ* hybridization that utilizes isothermal loop amplification reactions to detect the presence of gene specific mRNA or DNA in tissue sections. LAMP (Loop-Mediated Isothermal Amplification) is a method that uses an isothermal strand-displacing DNA polymerization reaction to amplify a target gene through the formation of dumbbell or end-looped structures as substrates [9–11]. Four to six primers are designed to amplify a target DNA or RNA [9]. In the case of amplifying a target mRNA, the reaction begins when the inner reverse, or back, complementary primer, BIP, hybridizes to the target mRNA and initiates first strand cDNA synthesis via a reverse transcriptase enzyme (Fig 1A). This polymerization creates a new DNA-RNA hybrid product out of the initial target mRNA. From this point, target mRNA and DNA amplifications are similar. The outer reverse or back primer, B3, binds to the template and displaces the first strand during strand elongation (Fig 1B). This initially frees the 5’ end of the first strand which loops on itself through complementary binding of the B1c and B1 sequences. The forward inner primer, FIP, binds to the F2 complementary sequence either in the first strand cDNA or the reverse DNA strand and triggers DNA polymerization using a strand displacing DNA polymerase until the complimentary strand is completely displaced (Fig 1C). The product strand is displaced by synthesis initiated by an outer primer, F3, creating a self-hybridizing loop structure in the product due to the attachment of the 5’ extension of the forward inner primer, F1c, to its complimentary sequence in the product strand, F1 (Fig 1D). The annealing and displacement process is then repeated in the reverse direction in the non-looping end of the product creating a dumbbell structure. This dumbbell structure provides multiple synthesis initiation sites at the 3’ end of the open loop as well as in the forward primer regions (Fig 1D). These sites are used to start up the amplification process and give rise to longer and more complex concatemers with more initiation sites.

LAMP is an attractive alternative to commonly used *in situ* hybridization methods because it resolves many of the problems and limitations found in current, common protocols, particularly those used for plant gene detection. It is expected to produce high amplification of all genes or mRNAs, including single copy genes or genes with limited expression patterns. Since LAMP is said to amplify both DNA and RNA, it can be utilized to detect both the presence and absence of a gene in a genome, as well as gene expression patterns. Its isothermal single reaction process reduces the number of steps in the methodology, leading to a decrease in tissue degradation and loss of expressed mRNA as well as increasing the time efficiency of the protocol. Because it is an isothermal reaction, simple incubators can be used instead of specialized temperature cycling equipment. As the reactions use four, relatively short primers with six unique sequences that must be arranged in a strict spatial order, it is possible to design highly discriminating assays. Similarly, due to the expected high specificity of the reaction, we expect LAMP to also be applicable for multiplex reactions, leading it to be more effective than a typical *in situ* hybridization or FISH protocol.

The aim of this project is to develop a novel *in situ* hybridization protocol that uses LAMP to amplify hard-to-detect target sequences from DNA and mRNA. Our goal is to resolve the most common limitations of *in situ* hybridization techniques. Specifically, to improve the integrity of the materials, our approach will reduce the processing of samples. The protocol will utilize relatively low temperature reaction conditions, thereby obviating the need for specialized equipment, and will produce strong amplification, thereby increasing the sensitivity to low signal. To increase specificity and reducing background, LAMP will require binding of six unique sequences, and the use of fluorescent labels will allow for simultaneous detection of multiple target transcripts or genes. The development of this protocol will create a novel, reliable, and efficient way of amplifying and detecting the signal of genes with low expression patterns, and, thus, will produce a better way of visualizing differential gene expression.

## Material and methods

### Target genes and tissues

We selected three gene from cultivated spinach (*Spinacia oleracea*) for detection: *SpAMS* (*Spinacia ABORTED MICROSPORES*), *SpAG* (*Spinacia AGAMOUS*), and *SpPI* (*Spinacia PISTILLATA*). *SpAG* and *SpPI* have been previously studied, and their expression patterns and functions are fully described [12–14]. *SpAMS* has recently been identified in spinach (West and Golenberg, unpublished data). The *Arabidopsis AMS* homologue of *SpAMS* is a developmental gene responsible for the transcriptional regulation of pollen wall development [14]. It plays a role in both tapetum formation and dissolution as well as in microspore compartmentalization [14]. Expression of this gene is expected to be found in every stage of pollen development starting from the microsporangium stage all the way to the final stage when the pollen is released. Preliminary data indicates that *SpAMS* is male specific and limited to the inner lining of the anther, called the tapetum, the microsporangium, and the pollen wall [14].

*SpAG* and *SpPI* are both floral organ identity genes [12, 13]. *SpAG* is a C class floral organ identity gene that is expressed in both male and female floral meristems and is required for the formation of stamens and carpels, respectively [13]. Previous studies have found it to be expressed in early floral primordia and later regions responsible for reproductive primordia development^9^. As such, we expect detection of *SpAG* expression in both male and female spinach tissue, although in gender-specific patterns. *SpPI* is a B class floral organ identity gene and is only expressed in male spinach tissue [12]. We used *SpPI* primers only in the *in vitro* studies.

### *In vitro* LAMP

*In vitro* reactions were designed and executed to confirm that LAMP reactions can be used to amplify single genes from genomic DNA and total RNA. Initially a plasmid was used as a template to confirm the suitability of the gene-specific primer sets. Following this, genomic DNA and total RNA were tested as templates in comparable reactions. Primers were designed using PrimerExplorer V4 software (Copyright 1999–2005 FUJITSU LIMITED) (https://primerexplorer.jp/e/index.html) and are listed in Table 1. The primers were combined with the WarmStart^®^ LAMP 2X reagent (DNA & RNA) following the manufacturer’s protocol (New England Biolabs). A single condition reaction was made up of 15μl 2x LAMP Mix, 0.6μl 50x fluorescent dye, 6μl 10x primer mix containing 4 to 6 primers depending on the target sequence^12^, 7.4μl UV treated water, and 1μl of the target DNA or RNA. The 10x primer mix consisted of 16μM FIP, 16μM BIP, 2μM F3, 2μM B3, 4μM LF, and 4μM LB primers for the selected gene, diluted in UV treated water. The DNA used had a concentration of 200–300 ng/μl and the RNA used had a concentration of 100–150 ng/μl. The negative controls were made in a batch with the regular reaction, and then aliquoted to a separate tube, and contained no total RNA or genomic DNA templates. Each final 30μl reaction was aliquoted into three separate tubes containing 10 μl each for technical replicates. After preparation, each reaction was held at a constant temperature of 65°C for 45 minutes to 1 hour in a Stratagene Mx3000P RT-PCR machine. Fluorescent readings of dsDNA were taken every 20 sections. The quantitative results were visualized using MxPro—Mx3000P (Copyright 2007 Stratagene).

**Table 1:** List of primers.

Gene	Primer Name	Sequence
*SpAMS*	SpAMS.800FIP	5’—TCC ACT TCC ATT TTC TGA TGA TGC TCA ACA CGT AAT CAA GCA GGA- 3’
*SpAMS*	SpAMS.936BIP	5’—CGA CAG CAG AAT CAG AAT CAG AAT CCT ATA TGA TGG TTG TGA ATG TGT- 3’
*SpAMS*	SpAMS.782F3	5’—GGG ACT CGT TGC TAG AGG- 3’
*SpAMS*	SpAMS.959B3	5’—AGT GGT TGT GGA AAG GAC- 3’
*SpAMS*	SpAMS.910BLP	5’—CAG AAC CAC CAT TAC GGG CT- 3’
*SpAMS*	SpAMS.6FAM_910BLP	5’—[6FAM] CAG AAC CAC CAT TAC GGG CT- 3’
*SpAG*	SpAG.484FIP	5’—GCT TTG CTG AGC TCT TTC ATT TTC ATG AAC TGC ATA ACA ATA ACC AAT- 3’
*SpAG*	SpAG.609BIP	5’—ACC GGG TGG CAG TGA TTA TGC TTG GAA GTA ATT CCG TGA AT- 3’
*SpAG*	SpAG.465F3	5’—TCA TGC AGA AAA GGG AGA T- 3’
*SpAG*	SpAG.630B3	5’—TTG GTT GGA GAG CGT TTA- 3’
*SpAG*	SpAG.586BLP	5’—TCT TGT CCC CTC TCA GTC ATT- 3’
*SpAG*	SpAG.Cy5_586BLP	5’—[5Cy5] TCT TGT CCC CTC TCA GTC ATT- 3’
*SpPI*	SpPI.205FIP	5’—CAA CCT CTT ACC GGA AAT ATT CTG AAA AAT GCA TGC TTA CAA TAG CC- 3’
*SpPI*	SpPI.356BIP	5’—GCT AAG CAT GAG GAG CTG AAG ATC AAG TGC CTG AGT TCG A- 3’
*SpPI*	SpPI.183F3	5’—TGT TAT CTT TGC TAA CAA TGG C- 3’
*SpPI*	SpPI.374B3	5’—GAT GCA ATG TCC TCT CCA T- 3’
*SpPI*	SpPI.232FLP	5’—TCT CAA GTA TAT CTT CCA CCG GAG T- 3’
*SpPI*	SpPI.330BLP	5’—CAA GAA AGA AAA TGA CGA TAT GCG G- 3’

### *In situ* LAMP

LAMP reactions were carried out on slides with sections of young male and female spinach inflorescence tissue. Genes with known expression patterns as described above, *SpAMS* (*Spinacia Aborted Microspores*) and *SpAG* (*Spinacia AGAMOUS*), were targeted. For the *in situ* reactions, the tissue was first dewaxed and rehydrated as described below and hybridized overnight with the BIP primer for the relevant gene. Slides with the most visibly intact tissue were selected for the hybridization. The LAMP reaction was conducted directly on the hybridized slides using the same reaction composition and conditions as in the *in vitro* tests. The slides were incubated for one hour at 65°C in a sealed, high humidity container and results were visualized on a fluorescence detecting microscope.

#### Tissue embedding

Samples were dehydrated and embedded following Sather et. al. 2005 with minor changes. Inflorescence tissue was harvested and submerged in an excess of formaldehyde-acetic acid alcohol (FAA) solution. The tissue was degassed by vacuum for 2 minutes and gently agitated before the vacuum was slowly released. If the samples did not sink, they were agitated gently, and the vacuum step was repeated. After degassing, samples were incubated in FAA for 10 to 14 hours at 4°C. They then underwent multiple washes. First, they were washed 2 times for 5 minutes in 50% ethanol and then 2 times for 30 minutes in 50% ethanol. Then for 10 minutes at 60°C in increasing concentrations of ethanol (75%, 85%, 95%, 2x 100%). The samples were washed for 10 minutes at 60°C in EtOH:Histoclear solution of decreasing ratios (3:1, 1:1, 1:3). Then a 5 minute wash in Histoclear was done followed by a 10 minute wash in 1:1 Histoclear:Paraffin and finally by five 30 minute washes in Paraffin. Wax embedded tissue was sectioned into 8μm samples and mounted on TruBOND 380 slides (Electron Microscopy Sciences, Hatfield, PA).

#### Hybridization

Slides with tissue samples were washed 4 times for 5 minutes each in Histoclear at 50°C to remove the wax. Then they were washed 3 times for 2 minutes each in 100% ethanol, followed by 2-minute washes in ethanol of decreasing concentration (95%, 85%, 70%, 50%). Then they were washed for 2 minutes in DEPC water and finally for 2 minutes in 1x TBS. At this point the slides could be held overnight in 1x TBS at 4°C if needed. A digestive solution was prepared, consisting of 0.25μl Proteinase K solution (20ng/μl), 1.25μl Triton x-100, and 248.5μl 1x TBS, for each slide. The slides were flooded with the solution and placed in a humidity chamber with no cover slip for 20 minutes at 37°C. The slides were then washed 3 times for 5 minutes each in 1x TBS. A 1μM solution of the BIP probe for the selected gene/s was aliquoted out and denatured at 80°C for 2 minutes and then quenched on ice. The probe was diluted in a 1:50 ratio into ULTRAhyb^™^ Ultrasensitive Hybridization Buffer and applied onto the slides, 250μl per slide. The slides were placed into a preheated humidity chamber at 55°C for 1 hour. The temperature was then reduced to 40°C and the slides were left in the chamber for 4 hours to overnight. After overnight hybridization, the slides were washed at 40°C, first in 2x SSC twice for 15 minutes, then in 1x SSC for 15 minutes, and finally equilibrated in 1x TBS.

#### LAMP reaction

During the equilibration steps, 30μl of LAMP reaction mix were prepared for each slide. They contain 15μl 2x LAMP Mix, 9μl UV treated water, and a 6μl of 10x primer mix with the BIP primer being substituted for a fluorescent labeled one. *SpAMS* was labeled with 6-FAM^™^, which has a max excitation of 495nm and a max emission of 520nm, while *SpAG* was labeled with Cy5^™^, which has a max excitation of 678nm and a max emission of 694nm. For the negative control, the slides were hybridized but the reaction mix did not contain the LAMP enzyme mix. The reaction solution was applied to the slides and they were then placed in a sealed preheated humidity chamber for 45 minutes at 65°C. The reaction was quenched on ice and the slides were visualized using a fluorescent detection capable microscope. Cover slips were not used for the entire procedure to reduce potential degradation of the tissue. Leica Application Suite X (Copyright 2017 Leica Microsystems CMS GmbH) and NIS Elements Viewer 4.50 (Copyright 1991–2015 Laboratory Imaging) were used to capture and view the images. The protocol was adjusted for multiplex reactions with the reaction mix containing 15μl 2x LAMP Mix, 3μl of 20x *SpAMS* primer mix, 3μl of 20x *SpAG* primer mix, and 9μl of UV treated water. During visualization of the slides, the Cy5^™^ labeled *SpAG* was screened first so as to not create a residual fluorescence after the filter cubes were switched.

For colorimetric detection, the reaction conditions were identical except for the following steps. Regular BIP primers were used instead of fluorescent varieties, the UV water was reduced to 8μl and 1μl of DIG-11-dUTP (25nmol) was added to the LAMP reaction mix. Additionally, the colorimetric assay can be accomplished without the preceding overnight hybridization step. Following the tissue section dewaxing and digestions processes noted above 30μl LAMP reaction mix consisting of 15μl 2x LAMP mix, 6μl 10x gene specific primer mix, 8μl UV treated water, and 1μl DIG-11-dUTP (25nmol) was then applied to the tissue sections dropwise to ensure adequate coverage. Slides were placed in a humidity chamber without a coverslip and the chamber incubated at 65°C for 45min. Slides were then washed 2 times for 5 minutes each in 1x MBS, flooded with blocking buffer and incubated for 1 hour at room temperature with gentle agitation. The slides were then placed into a humidity chamber and 250μl of blocking buffer containing a 1:5000 dilution of alkaline phosphatase conjugated anti-DIG anti-body was applied, the chamber sealed and incubated at room temperature for 1 hour. Slides were then washed four times for 10 minutes each and 250μl NBT/BCIP substrate was applied and incubated at room temperature for 20 to 25 minutes. The reaction was stopped by carefully flooding the slides with deionized water. A coverslip was applied with an aqueous mounting solution and tissue sections observed with a compound microscope.

A stepwise protocol is available on protocols.io at http://dx.doi.org/10.17504/protocols.io.57cg9iw.

### *In situ* hybridization

Tissue fixation, embedding, sectioning, and slide preparation were executed as described above. Following dewaxing, rehydration, and proteinase K treatment, sections were hybridized with digoxygenin-labeled, anti-sense *SpAMS* probes in ULTRAhyb buffer (Invitrogen) overnight at 50°C, followed by blocking with 1% non-fat milk solution and detection with alkaline phosphatase conjugated anti-DIG antibody and NBT/BCIP (Roche). The detection reaction was conducted at 37°C for one hour to overnight until a signal could be detected.

## Results

### *In vitro* LAMP amplifications of transcription factors from RNA and gDNA templates are rapid, robust, and specific

**Fig 2:**
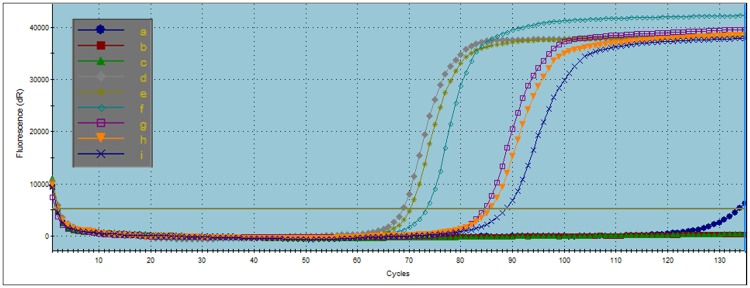
LAMP can be used with total RNA and genomic DNA. Four primers were designed to amplify the spinach *SpAMS* gene. Lines a-c) Negative controls. Lines d-f) Genomic DNA template. Lines g-i) Total RNA template. Cycles represent 20-second interval when double stranded DNA is measured. The amplification with the genomic DNA template initiated after approximately 22 minutes. The amplification with the total RNA template initiated after approximately 27 minutes. Both reactions were completed in approximately 6.6 minutes.

To verify the robustness of the LAMP reaction and to test the efficiency of the primers to uniquely amplify selected genes from both genomic DNA and total RNA, we ran replicate LAMP reactions *in vitro* on a Stratagene Mx3000P RT-PCR machine. The target genes, *SpAG*, *SpPI*, and *SpAMS*, all encode for transcription factors that are expressed in male and/or female inflorescence tissue in spinach [12, 13, 15]. The LAMP reactions for *SpAMS* were executed using the five primers listed in Table 1. Both genomic DNA and total RNA template reactions were tested in the following experiment. The negative control reactions contained all reagents except for template DNA or RNA (Fig 2, lines a-c). No amplification products were detected in the first 45 minutes, although one replicate reaction started to show some product after 45 minutes. The replicate reactions using genomic DNA as a template to test for *SpAMS* expression started to register amplification at approximately 22 minutes and plateaued within 6 minutes (Fig 2, lines d-f). Similarly, the amplification reactions using total RNA as template started to show amplification at approximately 26.6 minutes and reached a plateau within 6.6 minutes (Fig 2, lines g-j).

**Fig 3:**
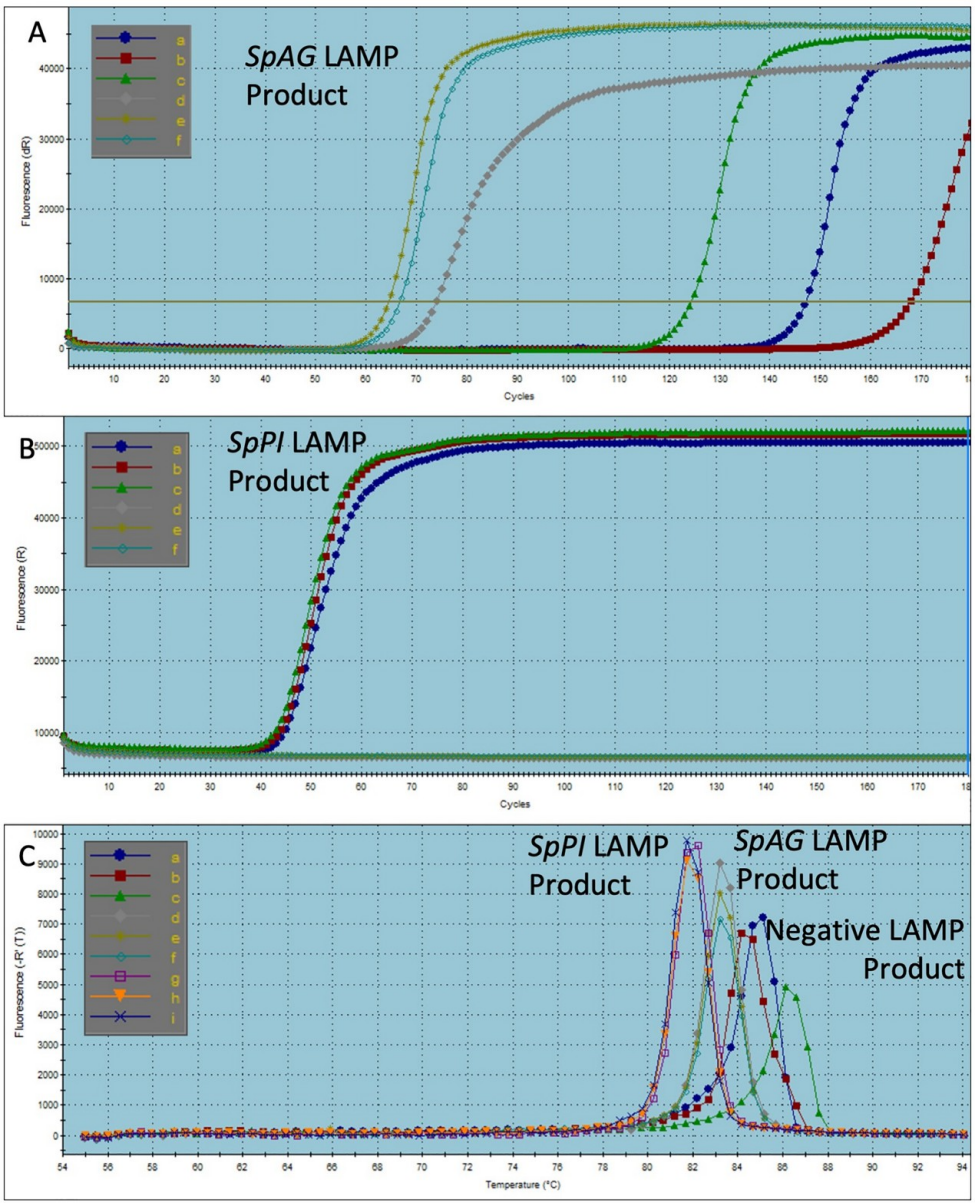
Gene specific products can be efficiently amplified using four to six primers. A. Five primers were used for *SpAG* amplification. The amplification of the *SpAG* fragment from total RNA began after approximately 18.5 minutes and was completed in about 6.6 minutes. No template replicates (lines a-c) showed evidence of late amplicon production. B. *SpPI* LAMP from total RNA using six primers demonstrated measurable amplifications after approximately 13 minutes and was completed in about 6.7 minutes. No products appeared in the no template controls (lines d-f) after an hour. C. Melting curve analysis of *SpAG*, *SpPI*, and no template control products with *SpAG* primers demonstrated unique melting point temperatures for each specific gene product, and multiple different melting points for negative control products indicating non-specific, non-repetitive amplicons.

The amplification reactions targeted the following two genes produced similar results. The reactions for *SpAG* utilized five primers (Table 1) and used only total RNA as template. The amplification of *SpAG* began after approximately 18.5 minutes and was completed in 6.6 minutes (Fig 3A, lines d-f). To assay for the repetition of false positive amplification products, the *SpAG* reactions were allowed to continue and be monitored for over one hour. Products were detected in the negative control replicates after approximately 45, 48, and 55 minutes (Fig 3A, lines a-c). As each reaction was aliquoted from one initial batch, the different amplification start times indicate random amplifications rather than amplifications of a uniform source contamination.

To confirm the rate of amplification and the time of appearance of spurious negative control products, reactions testing for the third target gene, *SpPI*, again using only total RNA as a template, were also extended for one hour. The *SpPI* amplification began at around 13 minutes and was completed in 6.7 minutes (Fig 3B, lines a-c). The negative controls produced no amplification after one hour (Fig 3B, lines d-f). The melting points of the *SpAG*, *SpPI*, and *SpAG* negative control products were tested to verify the integrity of the amplicons. The melting point of the gene specific amplified product were consistent across all replicates (Fig 3C, lines d-i), although differing, as expected, between the two genes. These indicate that the amplification products were unique and uniform in each reaction. In contrast, the late-appearing amplification product in the *SpAG* negative controls produced random, inconsistent melting points different from that of the positive results (Fig 3C, lines a-c).

Overall, the results of *in vitro* amplification show that all templates were successfully amplified using our designed primer sets. The amplification for each gene was consistent and robust across all replicates. All positive reactions reached a plateau well before product artifacts occurred in the negative controls. As such, it appears that background, false amplification will not generate significant heterogeneity in targeted amplifications provided that appropriate care is taken to avoid contamination.

### *In situ* fluorescent LAMP amplifications are highly sensitive and can be multiplexed for simultaneous gene expression detection

**Fig 4:**
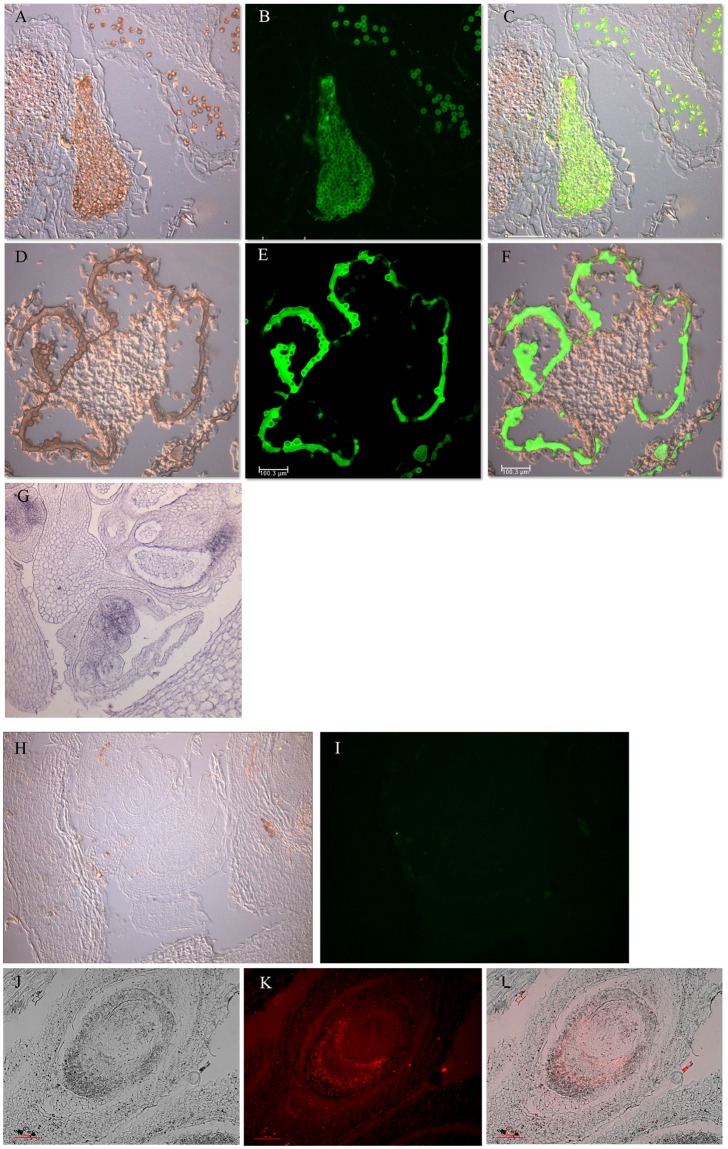
Single target LAMP produces robust *in situ* amplification. A-F. *SpAMS* expression (green) detected in male inflorescence tissue. A-C. Early anther showing pollen mother cells. A. Bright field. B. Green fluorescence. C. Overlay. D-F. *SpAMS* expression in post pollen release anther tissue. D. Bright field. E. Green fluorescence. F. Overlay. G. *In* situ hybridization using *SpAMS* antisense probe. Tapetal layers, microsporangial tissue, and vasculature tissue are stained blue. H-I *SpAMS* LAMP on female inflorescence tissue. H. Bright field. I. Green fluorescence. J-L. *SpAG* expression (red) in female inflorescence tissue. J. Bright field. K. Red fluorescence. L. Overlay.

After establishing the efficacy of the LAMP reactions specifically on RNA templates, we applied single target gene LAMP reactions to plant tissue sections on glass slides. Rehydrated tissue sections were hybridized overnight using the gene specific (*SpAMS*, *SpAG*) BIP primers. Following a wash of the hybridization solution, LAMP reactions were applied directly to the slides using the same reaction composition with the exception of the replacement of the original back loop primers with fluorescently labeled primers (Table 1). Based on the behavior of the *in vitro* reactions, the reactions were run for 45 minutes at 65°C. Negative controls using LAMP reagents without primers and primers without the LAMP enzyme mix were used to detect for non-specific autofluorescence and for non-specific binding of the fluorescent primers, respectively. The results of *in situ* LAMP reactions for the genes *SpAMS* and *SpAG* are displayed in Fig 4. *SpAMS* is a spinach male specific gene that is expected to be expressed in male microsporangial tissue and tapetal tissue [14]. The BLP primer for this gene was labeled with a 6-FAM^™^ flourescent label that flouresces green. After amplification, the gene was confirmed to be expressed in male microsporangia as well as high expression in tapetal tissue and high expression in pollen (Fig 4C). The positive reactions on female tissue showed little to no amplification (Fig 4I), thus confirming the tissue specificity of the reaction. The negative controls for both male and female tissue showed no expression.

For a comparison with standard *in situ* hybridization procedures, we hybridized a *SpAMS* anti-sense probe to male inflorescence tissue. The hybridization was executed overnihgt, then standard washing, blocking, conjugate binding, and detection steps were completed. *SpAMS* display weak, but discernable staining in the tapetal and microsporangial tissue (Fig 4G). These results confirm that the *in situ* LAMP and standard *in situ* hybridization techniques detect identical spatial expression patterns, but with higher sensitivity in the *in situ* LAMP procedures.

To contrast the male specific patterns of *SpAMS*, we challenged both male and female tissue for *SpAG* amplification. This gene is expected to be expressed in early primordia of male and female flowers as well as in the ovaries of young female flowers and stamens in male flowers [13]. The BLP primer for this gene was labeled with a Cy5^™^ flourescent label which flouresces with a red light. Positive flourescence was located was the inner tissue ring of a female ovary, the nucellus (Fig 4I and 4J). We were also able to detect expression of *SpAG* in male anthers before the formation of locules as reported previously. Together with the *SpAMS* detection, these results confirm that fluorescently labeled LAMP can be used to detect gene specific expression reliably.

**Fig 5:**
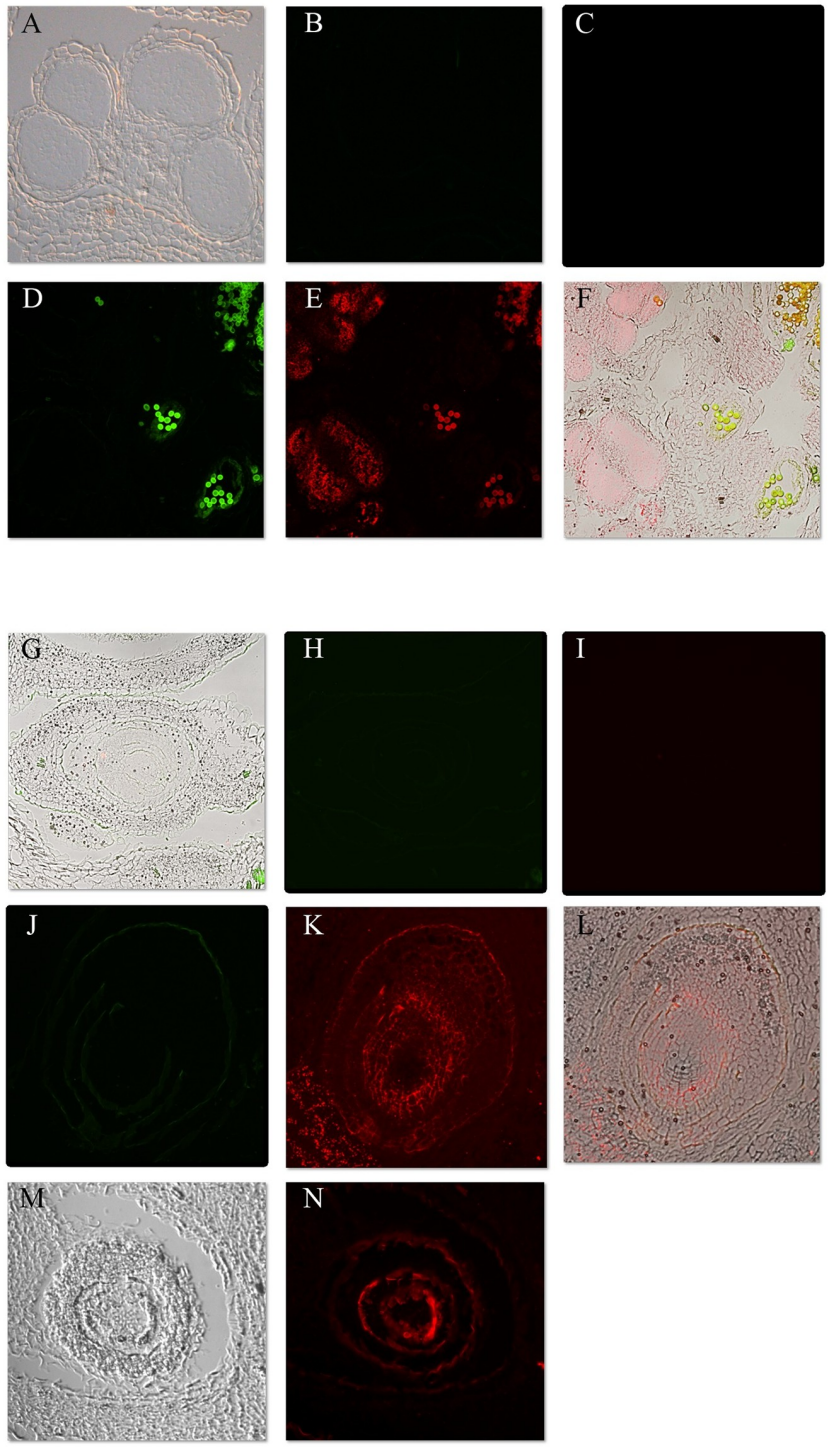
Multiplex LAMP produces alternative *in situ* amplification. *In situ* LAMP reactions were assembled with complete sets of *SpAMS* and *SpAG* primers. A-F. Male spinach inflorescence tissue. A-C. No enzyme negative control. A. Bright field. B. Green fluorescence. C. Red fluorescence. D-F. *SpAMS* (green) and *SpAG* (red) expression in male inflorescence tissue. D. Green fluorescence detected weak *SpAMS* in early tapetum. E. Red fluorescence detected *SpAG* expression in early microsporangial tissue. F. Overlay. G-N Female spinach inflorescence tissue. G-I. No enzyme negative control. G. Bright field. H. Green fluorescence. I. Red fluorescence. J-K *SpAMS* (green) and *SpAG* (red) expression in female inflorescence tissue. J. No detectable *SpAMS* under green fluorescence. K. Red fluorescence detected *SpAG* expression in nucellus. L Overlay bright field and red fluorescence. M-N. Mid-stage ovule. M. Bright field. N. Strong *SpAG* detection in nucellus under red fluorescence. Scale bar equals 100μM.

Given that the gene specific amplification is driven by the unique primer sequences, and that each primer set was differentially labeled, we wished to test whether replicable and reliable *in situ* detection can be achieved simultaneous for more than a single gene. Complete primer sets for both *SpAG* and *SpAMS*. fluorescently labled as described above, were used in a single LAMP reaction mix. Slides with male or female floral tissue were hybridized overningt using both BIP primers. The reaction mix with both primer sets was applied to each slide following hybridization wash. The multiplex LAMP reactions produced similar results for both genes that were found in the previous single gene reactions. Strong, non-overlapping signals from *SpAMS* (green) and *SpAG* (red) were detected in male tissue (Fig 5F). There was no significant background. In the female tissues, no *SpAMS* expression was detected, while strong *SpAG* expression was found in nucellar tissue as found in the single gene LAMP reactions (Fig 5K and 5N). The negative controls showed no amplification for either set of slides (Fig 5A–5C and 5G–5I). Thus, the multiplex reactions produced high fideltiy, gene specific and gender specific detection with no background.

### Colorimetric *in situ* LAMP amplification can be used for fast and less expensive detection of single genes

**Fig 6:**
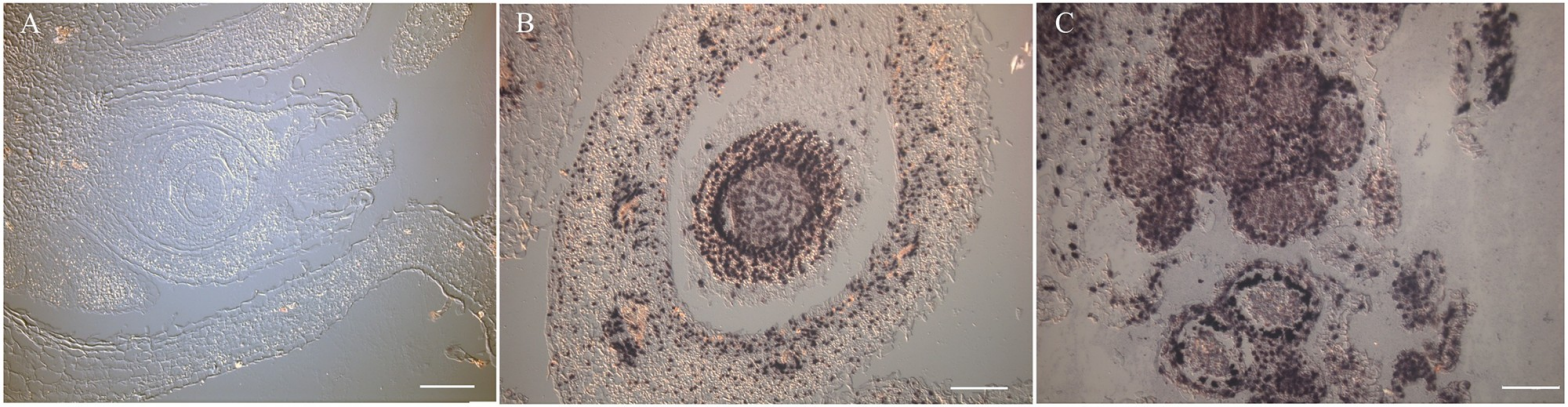
NBT/BCIP detection of *SpAG* expression. A. Female flower no enzyme negative control. B. *SpAG* expression in female flower tissue. C. *SpAG* expression in male flower tissue.

Fluorescent labeled primers that are required for multiplex LAMP *in situ* hybridizations are relatively expensive and are unnecessary for single gene *in situ* studies. To reduce the cost of such assays, we wished to modify our protocol to utilize standard alkaline phosphatase detection techniques. *SpAG* LAMP reactions were prepared with unlabeled primer sets but with 0.025μM digoxygenin-11-dUTP added. Rehydrated tissue sections were alternatively treated with overnight hybridization as previously described or no hybridization, and then applied with the digoxygenin containing LAMP reactions. Following a 45 minute incubation at 65°C and subsequent washes and blocking, the slides were treated with anti-digoxygenin-AP conjugates following standard techniques. An NBT solution was then applied and the slide was incubated at room temperature for 20 to 25 minutes. The reactions were stopped by washing in deionized water. Strong blue/black deposition was detected in both male and female floral tissue with low background (Fig 6B and 6C). The expression patterns followed previously published patterns and matched the patterns detected using the fluorescent labeled primers shown above. There were no consistent differences between slides with or without prior overnight hybridization. This step appears to be optional. The negative control showed no background staining (Fig 6A). These results confirm that incorporation of the digoxygenin labeled dUTP provides a rapid alternative protocol for *in situ* hybridization and requires less manual handling of the tissue sections on the slides.

## Discussion

Loop-Mediated Isothermal Amplification or LAMP is now recognized as a robust method for DNA replication. Its dependence on the correct spatial hybridization of six independent sequences oriented in four oligonucleotide primers results in high specificity. As such, it is becoming a widely used tool in parasitology, bacteriology, and histology for the detection of diagnostic targets [16–21]. LAMP is particularly advantageous as it is rapid and can be applied to aqueous samples. As it is an isothernal reaction with a relatively low optimal reaction temperature of 65°C, simple incubation devises can be used, thus making LAMP a potentially powerful tool for field studies. Detection of positive reactions in solution by simple assays provides a rapid readout [22–24].

In contrast to *in vitro* assays, *in situ* hybridization detects the presence of specific nucleic acid sequences, most commonly specific mRNA molecules, in their true histological, morphological and developmental contexts. Thus, *in situ* hybridization assays provide fine scale spatial and temporal information that is largely unobtainable from *in vitro* procedures. However, *in situ* hybridization procedures are inherently difficult to execute and typically suffer from degradation of sample tissues due to excessive handling, having limited sensitivity to genes with low expression, having reduced specificity and high background, and being unable to produce multiple gene detections [1–5, 7, 8]. Here we have shown that implementation of LAMP as a novel modification to the standard *in situ* protocols resolves most of the inherent *in situ* hybridization limitations.

Our results have several important implications for using LAMP as a standard *in situ* protocol. First, utilization of LAMP kits with reverse transcriptase activity allows for amplification of mRNA targets after the first strand cDNA is generated. Once the cDNA is generated, the actual amplification rate is identical to reactions using DNA as a template. Our *in vitro* experiments indicated completion of amplification within 6 to 7 minutes once amplification was detected. These results conform to previously published characterization of the LAMP [9]. Second, as the reactions plateau after such a short time, due to exhaustion of reagents and/or build up of pyrophosphates, off-target reactions, which, when they occur, begin after 45 minutes or an hour in negative control trials, cannot produce significant products. Thus, even in a complex reaction environment such as found in a tissue section, spurious products will be minimalized. Under conditions in which primer sets do not efficiently or specifically bind to and trigger amplification of target mRNA, false positives may occur. However, prior *in vitro* testing of primers should reduce false positive results. Alternate, primer sets targeting the same gene can be used to verify expression patterns. Third, LAMP reactions are driven by a minimum of six hybridization points (noted as F1, F2, F3, B1, B2, B3 in Fig 1) compared to two hybridization points in PCR. Additionally, the spatial orientation of the six is more restrictive than that of PCR primers. These requirements in primer design greatly reduce the potential for off-target signals.

Together these aspects of the amplification bias the *in situ* hybridizations to produce strong signal with weak background, and low probability of cross interaction of independent primer sets. The use of multiplexed primer sets targeting *SpAMS* and *SpAG* produced clear, non-overlapping signals in male spinach inflorescence tissue as expected (Fig 5D–5F), whereas, the same primer sets detected only *SpAG* in the female tissue where *SpAMS* is not expressed (Fig 5J–5L). The same high signal, low background was achieved when using digoxygenin incorporation and detection (Fig 6).

Prior studies have utilized specific aspects of our LAMP *in situ* protocol. Fluorophores have been used extensively in immuno-staining and chromosome painting [6, 25] and labeled primers have been incorporated in *in situ* PCR [26]. Meng et al. [27] reported using a fluorophore streptavidin conjugate to detect biotin labeled LAMP products of *rRNA* genes on *Chlamys farreri* chromosome preparations, and Gangoli et al. [28] utilized LAMP on pathology CHIP slides. Similarly, multiplexed LAMP reactions have been reported to detect products with different melting points in *in vitro* assays[19].

The use of multiplexed, fluorescent LAMP reactions for complex tissue analysis or the single-gene NBT/BCIP protocols presented here, however, should be seen as a significant new technique. The reactions significantly reduce the amount of time and handling during the detection process leading to improved sample integrity. The gene-specific amplification results in strong signal and low background. The use of unique fluorophores on primers permits simultaneous detection of multiple mRNAs in a single tissue, thereby producing a contemporaneous picture of genes during development or pathogenesis. Further simple modifications of this protocol such as labeling primers with biotin or digoxygenin followed by anti-digoxygenin and streptavidin coupled fluorophores may allow simultaneous expression analysis of multiple genes at a reduced cost by adding greater flexibility. Given its robust nature, we expect that *in situ* LAMP will become a powerful new tool for cell and developmental biology, pathology, cancer biology, and molecular ecology.
